# Internet-Connected Cortical Organoids for Project-Based Stem Cell and Neuroscience Education

**DOI:** 10.1523/ENEURO.0308-23.2023

**Published:** 2023-12-11

**Authors:** Matthew A. T. Elliott, Hunter E. Schweiger, Ash Robbins, Samira Vera-Choqqueccota, Drew Ehrlich, Sebastian Hernandez, Kateryna Voitiuk, Jinghui Geng, Jess L. Sevetson, Cordero Core, Yohei M. Rosen, Mircea Teodorescu, Nico O. Wagner, David Haussler, Mohammed A. Mostajo-Radji

**Affiliations:** 1 Genomics Institute, University of California Santa Cruz, Santa Cruz, CA 95060; 2 Live Cell Biotechnology Discovery Lab, University of California Santa Cruz, Santa Cruz, CA 95060; 3 Department of Biomolecular Engineering, University of California Santa Cruz, Santa Cruz, CA 95060; 4 Department of Molecular, Cell and Developmental Biology, University of California Santa Cruz, Santa Cruz, CA 95060; 5 Department of Electrical and Computer Engineering, University of California Santa Cruz, Santa Cruz, CA 95060; 6 Department of Computational Media, University of California Santa Cruz, Santa Cruz, CA 95060; 7 Scientific Software Engineering Center, eScience Institute, University of Washington, Seattle, WA 98195; 8 College of Arts and Sciences, University of San Francisco, San Francisco, CA 94117

**Keywords:** brain organoids, internet-of-things, neuroscience, organoids, stem cells, STEM education

## Abstract

The introduction of Internet-connected technologies to the classroom has the potential to revolutionize STEM education by allowing students to perform experiments in complex models that are unattainable in traditional teaching laboratories. By connecting laboratory equipment to the cloud, we introduce students to experimentation in pluripotent stem cell (PSC)-derived cortical organoids in two different settings: using microscopy to monitor organoid growth in an introductory tissue culture course and using high-density (HD) multielectrode arrays (MEAs) to perform neuronal stimulation and recording in an advanced neuroscience mathematics course. We demonstrate that this approach develops interest in stem cell and neuroscience in the students of both courses. All together, we propose cloud technologies as an effective and scalable approach for complex project-based university training.

## Significance Statement

The use of stem cell-derived cortical organoid models in academia and biotechnology has drastically increased in recent years. Given these trends, there is a critical need for students to be trained in organoid culture, differentiation, and analysis. To date, education curricula that focus on organoids are theoretical. Taking advantage of cloud technologies, such as Internet-connected microscopes and multielectrode arrays (MEAs), we propose approaches to introduce students to cortical organoids using live experiments. We show that these approaches develop interest in the field and prospects in students in biology and other STEM disciplines, such as mathematics and computer science.

## Introduction

Pluripotent stem cell (PSC)-derived 3D cortical organoids are transforming the study of human development, disease, and evolution (Kadoshima et al., 2013; Pollen et al., 2019; Mostajo-Radji et al., 2020; Velasco et al., 2020; Nowakowski and Salama, 2022; Paulsen et al., 2022). Organoids model several aspects of corticogenesis, including the emergence of cell types, such as neuronal progenitors, projection neurons, interneurons, astrocytes, and other glia cell types (Pollen et al., 2019; Velasco et al., 2020; Nowakowski and Salama, 2022; Paulsen et al., 2022), as well as formation and maturation of neuronal networks (Trujillo et al., 2019; Fair et al., 2020; Sharf et al., 2022; Cai et al., 2023).

The use of cortical organoids in academic research and biotechnology has drastically increased in recent years (Salick et al., 2021). For example, cortical organoids are used as tools for drug discovery (Salick et al., 2021), to study genetic mutations underlying disease (H. Wang, 2018; Paulsen et al., 2022), and to study infectious diseases that affect the brain, such as Zika and COVID-19 (Garcez et al., 2016; Andrews et al., 2022). Given their applications and the trends in the use of these models in research and development, there is a growing need for students to be trained in stem cell culture and cortical organoid generation, manipulation, and analysis.

Tissue culture courses are challenging to implement because of the high costs of infrastructure and supplies associated with the course, as well as the danger of exposure of students to biohazardous materials (Bowey-Dellinger et al., 2017; Robinson et al., 2020). Indeed, the large majority of undergraduate biology students are not trained in cell culture during their education (Bowey-Dellinger et al., 2017). The students who are fortunate to learn tissue culture techniques are usually trained in either plant cell cultures (Siritunga et al., 2012; Harahap et al., 2019) or simple animal cell culture, such as growth and passage of established cell lines (Mozdziak et al., 2004; McIlrath et al., 2015; Bowey-Dellinger et al., 2017; Phelan and Szabo, 2019) or primary cells (Lemons, 2012; Burdo, 2013; Catlin et al., 2016; Haskew-Layton and Minkler, 2020). While remarkable, these approaches are not sufficient to familiarize students with increasingly complex models, such as PSC culture, differentiation, and formation of 3D models, including organoids.

Despite the high interest of students in the topic (Bajek and Drewa, 2011), classes that focus on stem cell use and applications are mostly theoretical (Pierret and Friedrichsen, 2009; Ferreira et al., 2019; W Sandoval et al., 2022; Perlin et al., 2023). Courses with a lab component usually take advantage of invertebrate organisms such as planaria worms (Accorsi et al., 2017; Ochoa et al., 2019) or hydra (Larouche et al., 2020). On the other hand, courses that integrate mammalian stem cell culture are limited to elementary models such as culture and rapid differentiation of primary multipotent stem cells derived from the bone marrow of rodents (Jin et al., 2018), not PSC-derived models such as complex tissues and organoids. These fail to meet the need for students trained in high-level biomedical cell models. Indeed, students are usually first exposed to PSC-derived models in special extracurricular undergraduate research experiences, such as internships (Halverson et al., 2009). These research experiences are disproportionately accessible to students from privileged backgrounds and institutions (Grineski et al., 2018). To create a diverse workforce in the stem cell field we must find alternative approaches that can effectively train underserved students (Hruska et al., 2022).

Internet-enabled technologies can be a powerful tool to overcome this gap. When laboratory equipment is connected to the cloud, scientists and students can easily access and manipulate these technologies remotely (Ly et al., 2021; Baudin et al., 2022a; Nagarathna et al., 2022; Parks et al., 2022). Moreover, this approach allows exceptional platform scalability, allowing dozens or hundreds of users simultaneously (Baudin et al., 2022b). In the classroom, Internet-enabled technologies have been used to drive project-based learning (PBL) approaches in a variety of settings, including the use of microscopes for remote observations of behaviors of model organisms and cell cultures (Hossain et al., 2016; Ly et al., 2021; Baudin et al., 2022b), the use of sensors to monitor environmental conditions (Tsybulsky and Sinai, 2022; Tabuenca et al., 2023), and even the use of lab-on-a-chip microfluidic devices to perform bacterial detection in water samples (Sano et al., 2023).

Here, we take advantage of Internet-enabled technologies to introduce cortical organoid PBL in two different university courses: an introductory tissue culture course, where students monitor the growth of organoids in the presence of different drugs, and an advanced neuroscience mathematics course, where students stimulate and record from cortical organoids using multielectrode arrays (MEAs). In both approaches, the students can perform PBL over several days. We showed that Internet-enabled PBL leads to a high level of interest in neuroscience and stem cell biology in the students in both courses. Altogether we propose that Internet-enabled PBL can become an effective approach to deliver complex concepts in stem cell and organoid culture.

## Materials and Methods

### Ethics statement

The University of California Santa Cruz Institutional Review Board (IRB) reviewed this work at the proposal stage and determined that it did not constitute human subject study. Therefore, the work was IRB exempt.

### Students

The students were part of two different courses. (1) The students who performed the remote microscopy experiments were undergraduate students enrolled in the Techniques in Tissue Culture course at the University of San Francisco. A total of 10 students was enrolled in this course. (2) The students who performed the electrophysiology experiments were enrolled in the BME118: Mathematics of the Mind course at the University of California Santa Cruz. A total of 24 students was enrolled in this course.

### Surveys

All surveys were performed using Google Forms. The students received the link to the survey from their instructors. All surveys were completely anonymous and we did not record any identifiable information from any of the students. All students were informed that the answers to the surveys were anonymous and would not influence their grades.

For the Techniques in Tissue Culture course, we performed one survey at the end of the course. All 10 students who were part of the course responded to this survey. For the Mathematics of the Mind course, we performed two surveys: one before the experiment, and one after the experiment. For the pre-experiment survey, 18 students responded. For the postexperiment survey, all 24 students responded.

### Statistics

All of the survey questions were statistically analyzed in Python to determine the significance of responses. Significance was primarily measured using a one-sample, two-tailed *t* test. The test determines in which survey questions student responses are statistically different from neutral. In order to have an analyzable distribution, we numerically labeled student responses, “strongly disagree” to “strongly agree,” from −2 to 2. Under the null hypothesis for the one-sample *t* test, student responses are neutral (mean 0). Of the 49 survey questions asked, 92% of them were statistically significant (*p*-value 0.05).

To check whether final outcomes were consistent between the two courses, postcourse comparisons between the Stem Cell Techniques and the Mathematics of the Mind were performed using Prism 9.5.1 (GraphPad Software). The students’ answers were converted to a 1–5 points scale. Comparisons were performed using unpaired two-sample *t* tests. Significance was considered when *p* ≤ 0.05. The results from all surveys compared showed now significance between the distributions, meaning that the surveyed results remained consistent between classes.

Given that we are working with discretized ordinal (ranked) data, the normality assumptions of the *t* test do not completely hold. In such instances, the Wilcoxon signed-rank test is commonly used. We performed a one-sample Wilcoxon signed-rank test with a median 0 null hypothesis. However, this nonparametric test was limited by the small sample size of our data, 10–24 students per survey. Other statistics that were measured for each survey question include the data’s mean, median, mode, standard deviation, variance, standard error, range, skew, and kurtosis.

A complete summary of each of the survey’s statistics, as well as the cumulative data used to generate them, can be found at the following link: https://github.com/braingeneers/IOT_Education_Stem_Cell_Paper/blob/main/Main_Code/survey_statistics.ipynb.

### Embryonic stem cell culture

All experiments were performed in the ES-E14TG2a mouse embryonic stem cell (ESC) line (ATCC CRL-1821). This line is derived from a male of the 129/Ola mouse strain. Mycoplasma testing confirmed lack of contamination.

ESCs were maintained on recombinant human protein vitronectin (Thermo Fisher Scientific #A14700) coated plates using mESC maintenance media containing Glasgow Minimum Essential Medium (Thermo Fisher Scientific #11710035), embryonic stem cell-qualified fetal bovine serum (Thermo Fisher Scientific #10439001), 0.1 mm MEM nonessential amino acids (Thermo Fisher Scientific #11140050), 1 mm sodium pyruvate (Millipore Sigma #S8636), 2 mm glutamax supplement (Thermo Fisher Scientific #35050061), 0.1 mm 2-mercaptoethanol (Millipore Sigma #M3148), and 0.05 mg/ml primocin (Invitrogen #ant-pm-05). mESC maintenance media were supplemented with 1000 units/ml of recombinant mouse leukemia inhibitory factor (Millipore Sigma #ESG1107), 1 μm PD0325901 (Stem Cell Technologies #72182), and 3 μm CHIR99021 (Stem Cell Technologies #72054). Media were changed every 2–3 d.

Vitronectin coating was incubated for 15 min at a concentration of 0.5 μg/ml dissolved in 1× PBS pH 7.4 (Thermo Fisher Scientific #70011044). Dissociation and cell passages were done using ReLeSR passaging reagent (Stem Cell Technologies #05872) according to the manufacturer’s instructions. Cell freezing was done in mFreSR cryopreservation medium (Stem Cell Technologies #05855) according to the manufacturer’s instructions.

### Cortical organoids generation

Mouse cortical organoids were grown as previously described by our group (Park et al., 2023). To generate cortical organoids, we clump-dissociated ESCs using ReLeSR and re-aggregated in lipidure-coated 96-well V-bottom plates at a density of 5000 cells per aggregate, in 150 μl of mESC maintenance media supplemented with Rho kinase inhibitor (Y-27632, 10 μm, Tocris #1254), 1 μm PD0325901 (Stem Cell Technologies #72182), 3 μm CHIR99021 (Stem Cell Technologies #72054; day −1).

After 1 day (day 0), we replaced the medium with cortical differentiation medium containing Glasgow minimum essential medium (Thermo Fisher Scientific #11710035), 10% knock-out serum replacement (Thermo Fisher Scientific #10828028), 0.1 mm MEM nonessential amino acids (Thermo Fisher Scientific #11140050), 1 mm sodium pyruvate (Millipore Sigma #S8636), 2 mm Glutamax supplement (Thermo Fisher Scientific #35050061), 0.1 mm 2-mercaptoethanol (Millipore Sigma #M3148), and 0.05 mg/ml Primocin (Invitrogen #ant-pm-05). Cortical differentiation medium was supplemented with Rho kinase inhibitor (Y-27632, 20 μm #1254), WNT inhibitor (IWR1-ε, 3 μm, Cayman Chemical #13659), and TGF-β inhibitor (SB431542, Tocris #1614, 5 μm, days 0–7). Media were changed every other day until day 7.

On day 7, organoids were transferred to ultra-low adhesion plates (Millipore Sigma #CLS3471) where media were aspirated and replaced with fresh neuronal differentiation media. The plate with organoids was put on an orbital shaker at 60 revolutions per minute. Neuronal differentiation medium contained DMEM: nutrient mixture F-12 with GlutaMAX supplement (Thermo Fisher Scientific #10565018), 1× N-2 supplement (Thermo Fisher Scientific #17502048), 1× chemically defined lipid concentrate (Thermo Fisher Scientific #11905031), and 0.05 mg/ml primocin (Invitrogen #ant-pm-05). Organoids were grown under 5% CO_2_ conditions. Medium was changed every 2–3 d.

On day 14 and onward, we added 5 μg/ml Heparin sodium salt from porcine intestinal mucosa (Millipore Sigma #H3149) and 0.5% v/v Matrigel growth factor reduced (GFR) basement membrane matrix, lactose dehydrogenase elevating virus (LDEV)-free (Matrigel GFR, Corning #354230) to the neuronal differentiation medium.

On day 21 and onward, we transferred the organoids to neuronal maturation media containing BrainPhys neuronal medium (Stem Cell Technologies #05790), 1× N-2 supplement, 1× chemically defined lipid concentrate (Thermo Fisher Scientific #11905031), 1× B-27 supplement (Thermo Fisher Scientific #17504044), 0.05 mg/ml primocin (Invitrogen #ant-pm-05), and 0.5% v/v Matrigel growth factor reduced (GFR) basement membrane matrix, LDEV-free.

### Microscopy experiments

The mouse cortical organoids were the basis of microscopy experiments. The microscope used to image these organoids is a variant of a previously described microscope (Baudin et al., 2022b), named the Streamscope. The Streamscope is a low cost and open hardware six-well microscope designed to perform simultaneous longitudinal imaging of cell culture in six separate wells. It produces timelapse sequences tracking morphologic changes in cell cultures. The device is constructed from a combination of off-the-shelf and custom-made components. The off-the-shelf components include a motor driver (Pololu Tic T825), a motor linear actuator module, an LED light panel, and six USB cameras (HBVCAM 5MP module with Omnivision 5640 sensor). The rest of the microscope is composed of parts which can be 3D printed or CNC milled in-house on consumer equipment or ordered from a third-party manufacturing service.

The organoids were transferred and adhered to a six-well plate (six-well Nunc Cell-Culture Treated Multidishes, Thermo Fisher Scientific #140685). Adhesion was accomplished by transferring 0.5 ml of Matrigel growth factor reduced (GFR) basement membrane matrix, LDEV-free (Matrigel GFR, Corning #354230) into a 1.5 ml Eppendorf Safe-Lock Tube (Thermo Fisher Scientific #22363204) kept on ice. The organoid was transferred into the Matrigel with a wide bore pipette, pipetting up and down gently five times to ensure maximal coating. The Matrigel-coated organoid was then transferred to the six-well plate and allowed to adhere for 10 min at 37°C before adding in fresh neuronal maturation media. For the experiments, we tested the effects of two drugs: 1 μm proapoptotic drug staurosporine (Thermo Fisher Scientific #NC1401148) and 1 μm DNA topoisomerase Type I inhibitor camptothecin (Thermo Fisher Scientific #501361118). In addition, the students had control organoids in which no drugs were added.

Two plates of organoids atop two Streamscopes were used for this experiment, totaling 12 potential wells worth of images. Images of each well were taken approximately every 60 s over a period of 5 d while the cultures were in the incubator. The images were streamed through YouTube, allowing the users to access the data in real time, as well as after the end of the experiment.

After the experiment was terminated, images from each condition were turned into a timelapse. The images for each well were converted into timelapses using Adobe Premiere. Images were computer analyzed for features such as organoid area.

### Organoid area measurements

To reduce the computational load on ImageJ, the software used to calculate the area of the organoids, the time lapse images were subsampled into sparser timelapse images. Starting with the 0-h time point, images of organoids were sampled every 6 h for 72 h total. By interpolating, adjusting threshold parameters in ImageJ and using the tracing tool, areas were determined for each organoid at all time points.

### Electrophysiology experiments

We plated mouse cortical organoids at day 22 on MaxOne high-density (HD) multielectrode arrays (Maxwell Biosystems #PSM). Before organoid plating, the multielectrode arrays were coated in two steps. First, we performed an overnight coating with 0.01% poly-L-ornithine (Millipore Sigma #P4957) at 37°C overnight. Then washed the plates three times with PBS. We then performed an overnight coating with 5 μg/ml mouse laminin (Fisher Scientific #CB40232) and 5 μg/ml human fibronectin (Fisher Scientific #CB40008) at 37°C.

After coating, we placed the organoids on the chip and removed excess media. The organoids were then incubated at 37°C for 20 min to promote attachment. We then added prewarmed BrainPhys neuronal medium supplemented with 20 ng/ml recombinant human brain-derived neurotrophic factor (Stem Cell Technologies #78005), 1× N-2 supplement (Thermo Fisher Scientific #17502048), 1× chemically defined lipid concentrate (Thermo Fisher Scientific #11905031), 1× B-27 supplement (Thermo Fisher Scientific #17504044), 0.05 mg/ml primocin (Invitrogen #ant-pm-05), and 0.5% v/v Matrigel growth factor reduced (GFR) basement membrane matrix, LDEV-free. We changed the media every 2–3 d.

Using the Maxwell Biosystems software, activity scans were performed every 2–3 d. We sampled signals from 1024 of the ∼26,000 electrodes at a time in a sweeping formation across the MEA at an interval of 30–45 s for each configuration. A base level of activity was measured by electrical events which crossed the 5-root mean square (rms) noise threshold. If activity was detected, 5- to 12-min recordings were taken using Maxwell Biosystems’ electrode selection algorithm which maximizes clusters of electrodes around hotspots of high relative firing rate. Neurons were identified by analyzing the neuronal footprint, the signal averaged waveform across a patch of multiple electrodes surrounding the potential neuron. Neurons chosen to be stimulated required detection of at least 10 threshold-crossing events. We chose stimulation channels as the channels that recorded the highest amplitude signals from the neuron to have the highest probability of evoking action potentials (Radivojevic et al., 2016).

### Data processing

Raw electrical data from the MEA was saved during each of the experiments in the hdf5 format. These data were spike-sorted into individual units (putative neurons) using Kilosort 2 (Pachitariu et al., 2023). This process filters the data, whitens (Pachitariu et al., 2023), then clusters neurons based on matching spike-waveform templates. For each identified neuron, this process outputs a list of spikes times together with the spatial location on the MEA. To validate the biological plausibility of the identified neurons, expert human curation was conducted through the open-source software Phy (https://github.com/cortex-lab/phy). Putative neural units are retained if they have key biological features such as a biologically consistent shape and duration for their action potential waveforms. Each experiment recording was individually processed with the same set of spike sorting parameters. Electric stimulation artifacts and noise were removed during the curation. These data were delivered to the students in Python Numpy format allowing easy subsampling.

### Electrophysiology experimental design

Mathematics of the Mind students were introduced to the idea of analyzing electrophysiological measurements by conducting analysis from a previous set of published electrophysiological recordings in human organoids (Sharf et al., 2022). The students were taught to use measures including spike-time tiling (Cutts and Eglen, 2014), correlation, interspike intervals, and latency distributions to characterize neural circuit behavior. After this initial homework assignment, students were asked to propose a circuit perturbation experiment using electrode-supplied stimulation on live tissue to augment the underlying neural circuitry, where they would analyze spontaneous activity before and after the perturbation. The setup of the experiment involved a 5-min baseline recording, a 5 min recording with student-designed stimulation, and a 15-min poststimulation recording. The students were specifically tasked with designing and coding their own stimulation paradigm to be executed during their experiment, and proposing a hypothesis for changes that would occur between the baseline and poststimulation recordings. They were encouraged to be creative.

### Stimulation programming

To make the task of stimulation paradigm design feasible and straightforward for the Mathematics of the Mind students, a simple application programming interface (API) was designed. The students were told that they could stimulate three neurons in any manner that the API enabled, and they could select the approximate distance of the three neurons as a proxy for connectivity. At a high level, the students craft sequences of stimulations using the API, and choose a frequency to iterate through these stimulation sequences. The API enables the creation of stimulation paradigms by three simple commands: “stim,” “delay,” and “next.” The “stim” command has three parameters: (1) the list of neurons to stimulate, (2) the amplitude in millivolts to stimulate, (3) the width of the biphasic pulse. Recommended values of 150 mV for the amplitude and 200 μs per phase for the biphasic pulse were given to students. The “delay” command has one parameter: the time to delay in milliseconds. It pauses the stimulation. The “next” command effectively ends the current stimulation sequence.

### Data availability

The consent given by the participants does not allow for open storage of data on an individual level in public repositories. Anonymized survey data are available on request by qualified scientists. Requests require a concept paper describing the purpose of data access, ethical approval at the applicant’s institution, and provision for secure data access. Cumulative data can be accessed in https://github.com/braingeneers/IOT_Education_Stem_Cell_Paper/blob/main/Main_Code/survey_statistics.ipynb.

## Results

### Internet-connected microscopy enables the incorporation of organoid modules in a tissue culture undergraduate course

PBL is an effective teaching approach for complex topics in biology, including teaching students who have traditionally underperformed in STEM (Ferreira et al., 2019; Petersen, 2021; Baudin et al., 2022b; Sano et al., 2023). We have previously shown that low-cost in-incubator Internet-connected microscopes can perform longitudinal imaging of cortical organoids and allow for the systematic tracking of organoid size and morphology over time (Ly et al., 2021), making them ideal tools to perform remote PBL training in the classroom (Baudin et al., 2022b).

As a proof of principle, we integrated an organoid tracking module within the Techniques in Tissue Culture course at the University of San Francisco, located at ∼125 km from the experimental site at the University of California Santa Cruz. This course has a strong hands-on component in which students learn basic techniques, including 2D culture of mammalian cancer cell lines. The students are exposed to the theory behind organoid generation, but the current curriculum does not have an organoid generation and culture experimental component. In this iteration of the course, 60% of the students were third year undergraduate students, 30% of them were fourth year undergraduate students and 10% of them were students in their fifth year or above.

To design a project of interest to the students, the professor who was leading the course had a brainstorming session with the students ahead of the experiment. In this session, the students were asked to nominate potential drugs that they thought would affect organoid growth. They nominated two drugs: staurosporine, a nonselective protein kinase inhibitor that is used in the research setting to induce apoptosis (Chae et al., 2000), and camptothecin, a selective DNA topoisomerase Type I inhibitor that has been used to treat different types of cancers (Martino et al., 2017).

**Figure 1.**
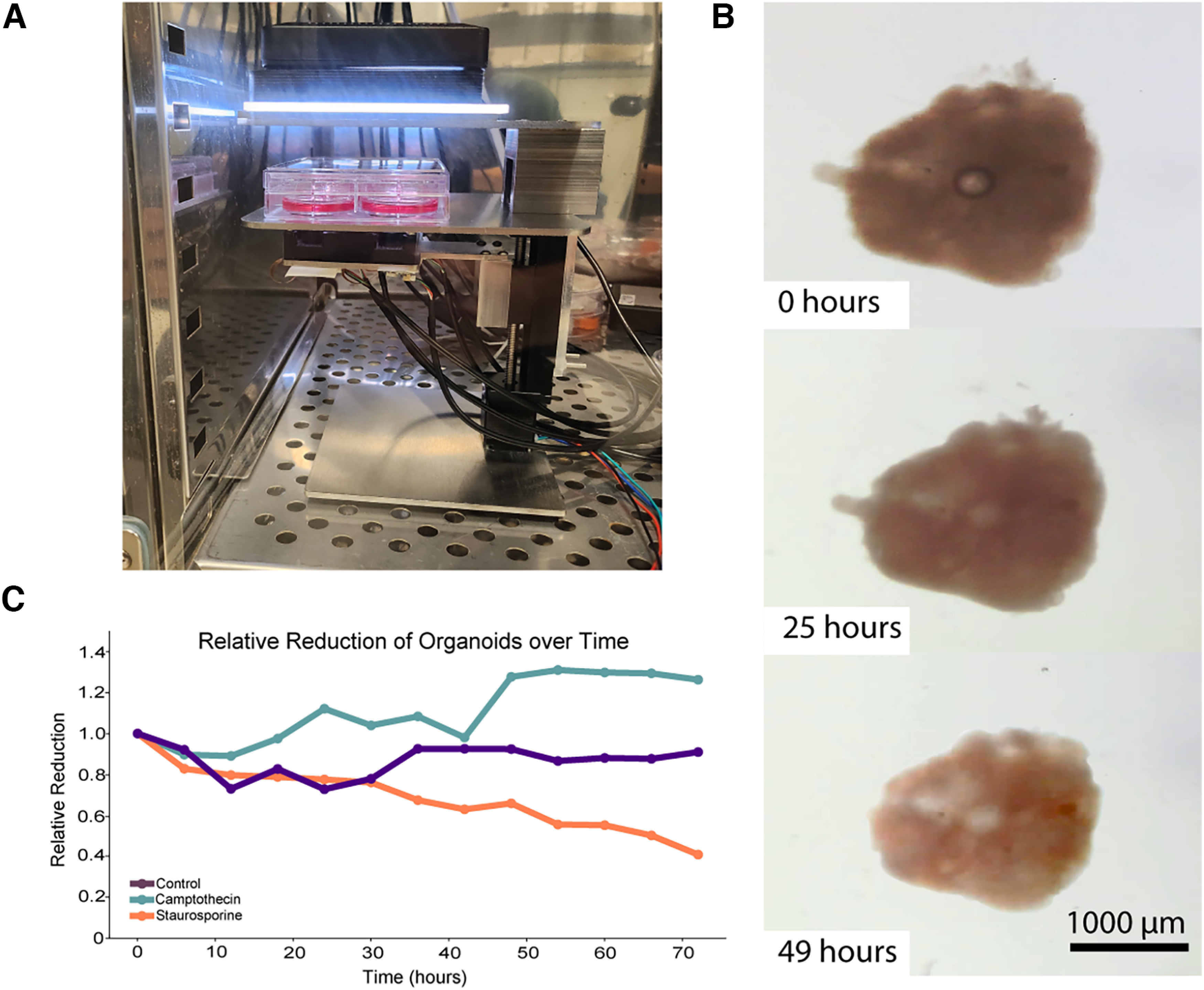
Internet-enabled microscopy enables longitudinal organoid tracking. ***A***, Experimental setup showing a Streamscope inside a tissue culture incubator tracking six organoids. ***B***, Example of an organoid exposed to the proapoptotic drug Staurosporine. ***C***, Example results obtained from a group of students calculating the maximal organoid area over 72 hours. For this experiment, each group of students measured three individual organoids, one of each condition.

To accommodate the students’ requested project, we continuously imaged mouse cortical organoids starting at differentiation day 26 using a Streamscope, an in-incubator low-cost microscope that has the ability to both record image stills and video, which are then streamed over the Internet through YouTube (Baudin et al., 2022b; Fig. 1*A*,*B*). The organoids were grown in the presence of either staurosporine or camptothecin. As control, organoids were grown without the presence of any drug. The Streamscope imaged organoids every 60 s for 3 d (Fig. 1*A*). This approach allowed us to constantly produce data and promote student engagement. The students were divided into groups of three to four and were asked to measure the maximal organoid area over time. They were then asked to discuss the effects of the drugs as a class. The students observed a reduction in organoid size in organoids that were treated with staurosporine (Fig. 1*B*). Consistent with the fact mouse cortical organoids of this age contain mostly postmitotic neurons (Eiraku et al., 2008; Kadoshima et al., 2013; Park et al., 2023), the students did not observe a reduction in organoid size in camptothecin-treated organoids (Fig. 1*C*). We therefore show that Internet-connected microscopes can effectively be used in the classroom setting to perform PBL approaches using organoid cultures.

### Remote cortical organoid culture leads to strong interest in stem cell topics

**Figure 2.**
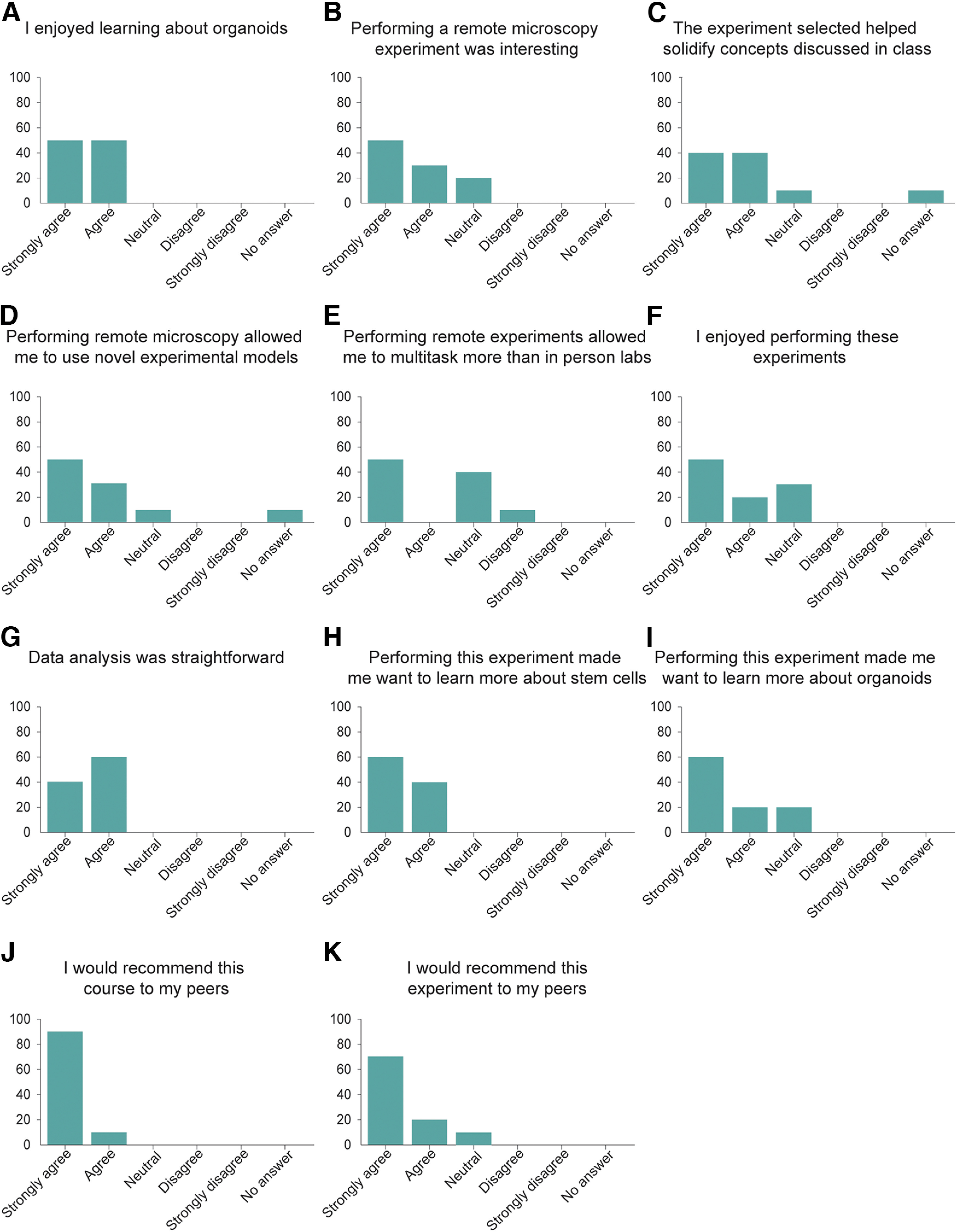
Students who perform remote cortical organoid culture report positive feelings and strong interest in stem cell topics. ***A–K***, Responses of the Techniques in Tissue Culture students to the postexperiment survey. *n* = 10 students. All of the 11 survey questions were statistically significant, using a one-sample two-tailed *t* test (*p*-value less or equal to 0.05). *y*-axis represents percent of students.

After the completion of the course, we surveyed the students to understand their satisfaction with the technology and the course, as well as their interest in topics related to stem cells and organoids. All students who were part of the course responded to the survey (Fig. 2).

To measure their satisfaction and interest, we asked the students their level of agreements with different statements. All students said that they enjoyed learning about organoids (Fig. 2*A*), and 80% of them thought that performing these experiments helped them solidify the concepts learned in class (Fig. 2*B*).

We then asked the students how they felt performing experiments using remote microscopy. We found that 80% of the students thought that performing remote microscopy experiments was interesting (Fig. 2*C*) and that it allowed them to use novel experimental models (Fig. 2*D*). Interestingly, only 50% of the students thought that they were able to multitask while performing these experiments (Fig. 2*E*), suggesting that for at least half of the students, performing remote experiments requires them to concentrate to similar levels than in person laboratories. Nevertheless, 70% of the students reported that they enjoyed performing these experiments (Fig. 2*F*) and all thought that data analysis was straightforward (Fig. 2*G*).

When asked how this project affected their interest in learning about stem cells and organoids, all the students reported that this project made them want to learn more about stem cells (Fig. 2*H*), while 80% of them reported that they want to learn more about organoids (Fig. 2*I*). Finally, we find that 90% of the students would recommend this project to their peers (Fig. 2*J*), while all students reported that they would recommend this class to others (Fig. 2*K*). Altogether, we conclude that Internet-enabled microscopy is an effective approach to introduce students to organoid culture, while increasing their interest in learning topics on stem cells and organoids.

### Electrophysiology software programs run on organoids in a neuroscience mathematics course

A landmark in neuronal maturation is the acquisition of electrophysiological properties (Fair et al., 2020). These properties follow a stereotypical cell type-specific identity (Ye et al., 2015; Cadwell et al., 2016; Zeng and Sanes, 2017; Gouwens et al., 2019). Given the great diversity of neurons in the cerebral cortex, it is expected that complex models, such as organoids, will have different electrophysiological profiles representative of their cell type composition (Passaro and Stice, 2021). Indeed, the emergence of complex networks in cortical organoids is an active area of research that is expected to continue growing over the next few years (Trujillo et al., 2019; Fair et al., 2020; Sharf et al., 2022; Cai et al., 2023).

One of the most exciting aspects of the emergence of neuronal circuits is that it provides scientists with the ability to study how neurons in organoids “learn” by adapting to stimuli provided to them (Zafeiriou et al., 2020). Neuronal plasticity can be evoked in cerebral organoids by sending electrophysiological stimulus patterns (Zafeiriou et al., 2020). These patterns are written using computer code. Therefore, an organoid’s neuronal circuitry can be “programmed” to produce different types of predictable neuronal responses.

To date, PBL-based teaching of electrophysiology concepts in the classroom have been limited to either simulation of single neurons using web-based apps (Demir, 2006; Cannon and Hammond, 2008; Formella-Zimmermann et al., 2022; Yamamoto et al., 2023) or the use of simple devices to activate and record neuromuscular electrophysiological signals in either invertebrates or the students’ own muscles (Marzullo and Gage, 2012; Ferreira et al., 2019; Hanzlick-Burton, et al., 2020; Ramadan and Ricoy, 2023). While remarkable, these experiments do not offer the capability to ask complex questions in circuit assembly and brain function. Specifically, students do not have the ability to program their own experiments, where they see how neuronal circuitry responds to their code. This is made possible using high-density multielectrode arrays (HD MEAs), in which thousands of electrodes can stimulate and record from a single circuit (Miccoli et al., 2019). For example, the MaxOne system from MaxWell Biosystems has been used to record circuit activity in cortical organoids through its 26,400 electrodes (Paulsen et al., 2022; Sharf et al., 2022). Yet, because of the high costs and experimental complexity associated with these experiments, HD MEAs have not been introduced in the classroom. We have previously developed an Internet-connected electrophysiology platform that allows the users to record from MEAs remotely (Voitiuk et al., 2021). This technology could become key for cloud-enabled PBL (Baudin et al., 2022b), in which students use experimental platforms that are not located at or owned by their own institutions.

**Figure 3.**
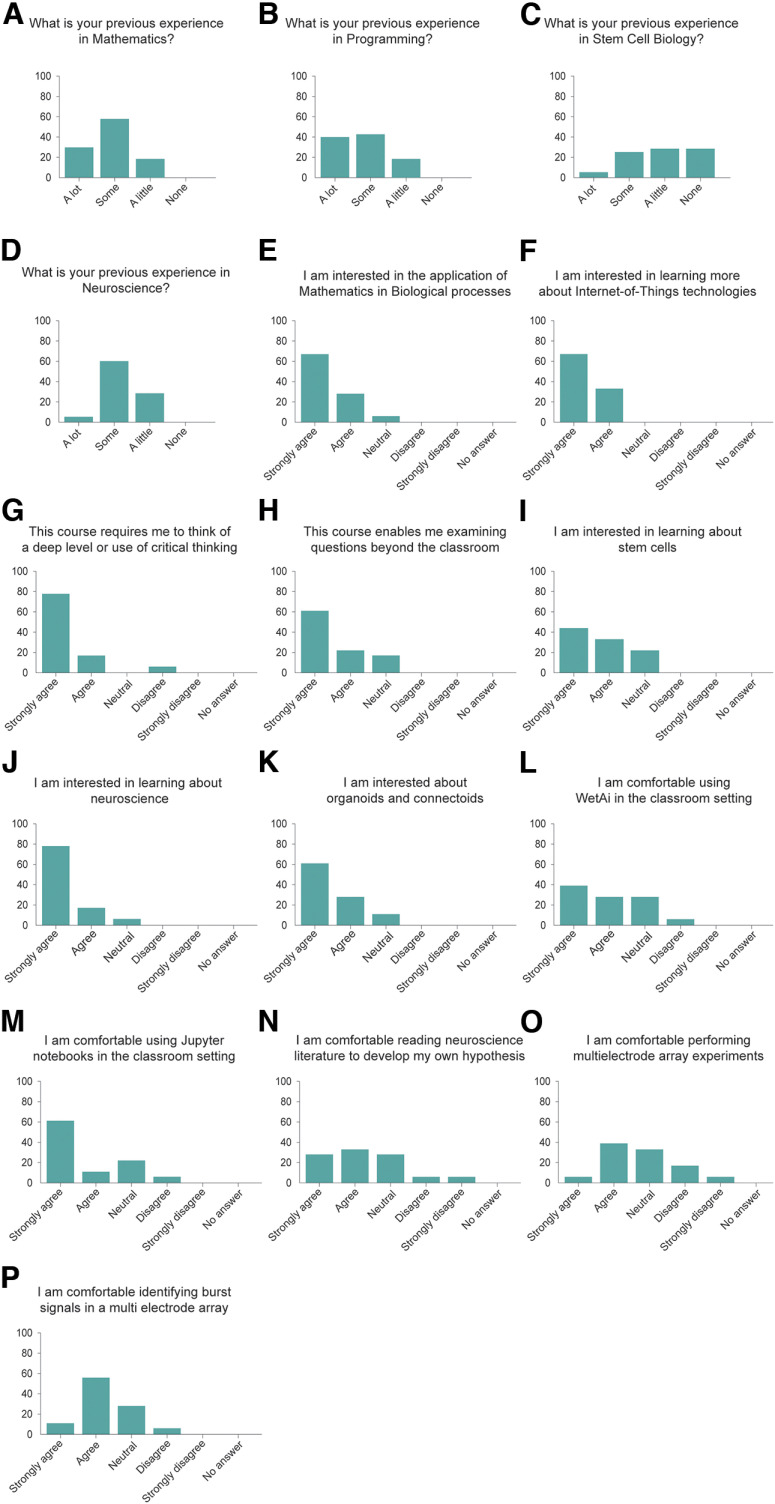
Students’ previous experience and reported interest in topics related to mathematics, neuroscience and stem cell biology. ***A–P***, Students’ answers to a survey assessing their level of experience and interest in the topics covered in the Mathematics of the Mind course. *n* = 18 students. A total of 14 of the 16 survey questions were statistically significant, using a one-sample two-tailed *t* test (*p*-value less or equal to 0.05). ***C*** and ***P*** were not significant. *y*-axis represents percent of students.

To deploy this technology for neuroscience education, we integrated it into the Mathematics of the Mind course at University of California Santa Cruz. This course is a mixed upper-level undergraduate and beginning graduate class where most enrolled students are from quantitative fields such as computer science, physics, applied mathematics, and engineering. Students were taught concepts and given supplementary readings within topics such as tetanus-induced potentiation dependent on connectivity (Jimbo et al., 1999), Hebbian theory (Hebb, 2005), spike-time dependent plasticity (Caporale and Dan, 2008) and homeostatic regulation (Harnack et al., 2015). For the students to properly construct experiments, they were introduced to applied electrophysiology techniques like spike sorting, a neurophysiology technique that allows neuronal spikes to be clustered based on similarities (Quiroga, 2012). To understand how performing live electrophysiological experiments impacted the students, we first surveyed them after the introduction of these concepts but before performing the experiments (Fig. 3). Of the 24 students enrolled in the class, 18 responded to the precourse survey. We found that the large majority (83.7%) of the students had previous experience in mathematics and computer programming (Fig. 3*A*,*B*). In contrast, 66.6% of the students reported having little to no previous stem cell biology experience (Fig. 3*C*) and 94% of the students reported having little to no previous experience with neuroscience (Fig. 3*D*).

We then asked the students questions related to their interest in the class and the topics discussed up to this point, and before the execution of live experiments. We found that 94.4% of the students reported that they are interested in the applications of mathematics to biological processes (Fig. 3*E*), and all of them (100%) wanted to learn about Internet-of-Things technologies (Fig. 3*F*). Similarly, 94.4% of the students reported that this course required them to think at a deep level or use critical thinking (Fig. 3*G*). In addition, 83.3% of the students thought that this course allowed them to examine questions that matter beyond the classroom (Fig. 3*H*). When asked about the biological side of the class, 77.7% of the students were interested in learning about stem cells (Fig. 3*I*) and 94.4% of them were interested in learning about neuroscience (Fig. 3*J*). Similarly, 88.8% of the students claimed to be interested in learning about “connectoids” (Fig. 3*K*), long-range connected organoids (Cullen et al., 2019; Kirihara et al., 2019; Adewole et al., 2021). When asked about the technologies to be used in the experiments, 66.6% of the students were comfortable using WetAI (Fig. 3*L*), our custom interface with the experiment (Baudin et al., 2022b), and 72.2% of the students claimed to be comfortable using Jupyter notebooks to perform experiments (Fig. 3*M*). We found that 61.1% of the students said they were comfortable reading neuroscience literature (Fig. 3*N*), while 44.4% of the students felt comfortable performing high-density MEA experiments at this time (Fig. 3*O*). In addition, 66.6% of the students reported that they would be comfortable identifying spike burst signals in high-density MEA data (Fig. 3*P*). Altogether, we observed that, consistent with their academic background, most students are more comfortable with the quantitative aspect of the class than with the biological section, although they demonstrate a high interest in learning topics in stem cell and neuroscience.

**Figure 4.**
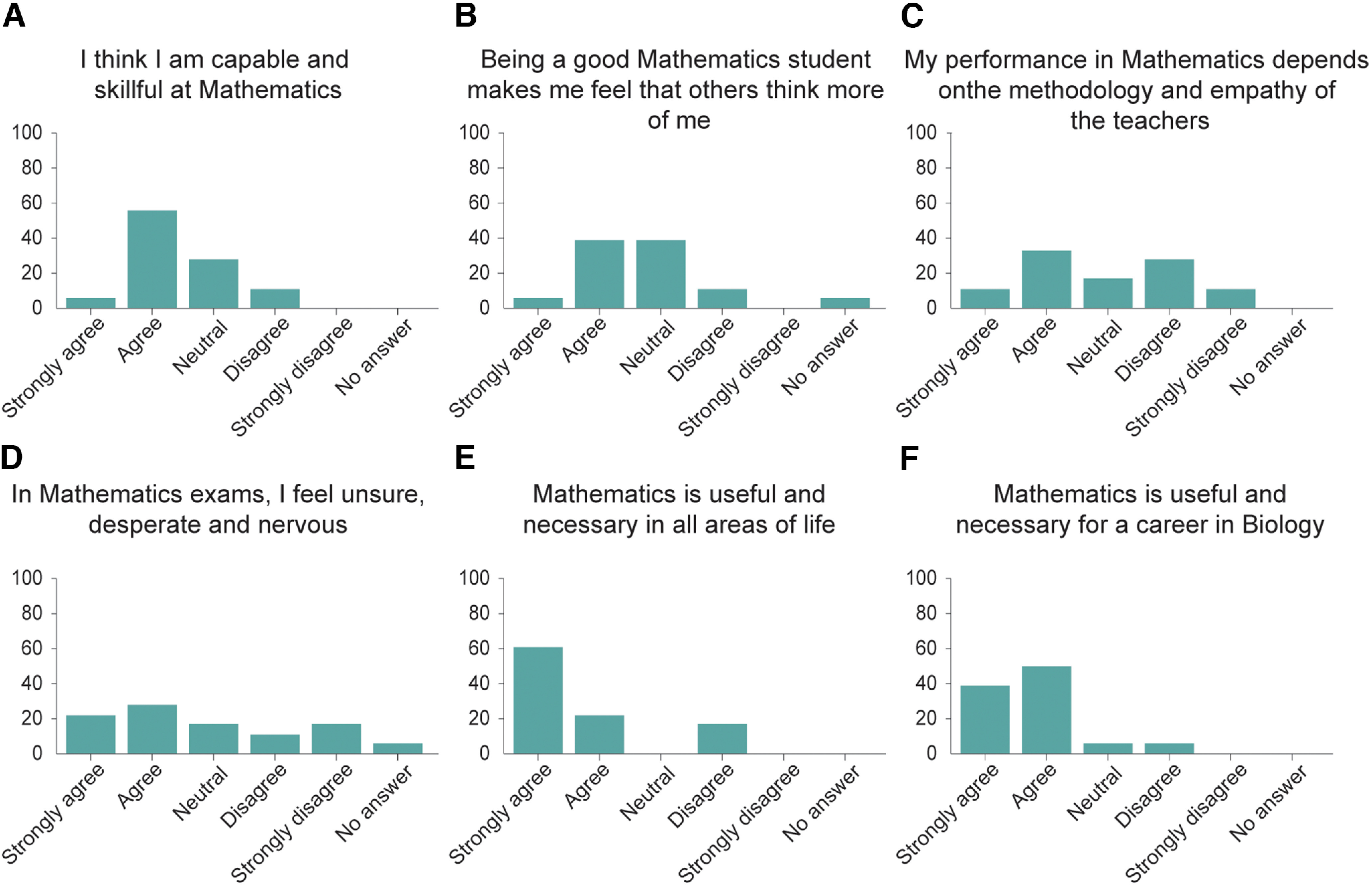
Mathematics self-concept of the students. ***A–F***, Students’ answers to a survey assessing their mathematics self-concept. *n* = 18 students. Four of the six survey questions were statistically significant, using a one-sample two-tailed *t* test (*p*-value less or equal to 0.05). ***C*** and ***D*** were not significant. *y*-axis represents percent of students.

Given the qualities of these students, we then asked them questions related to their mathematics self-concept, as this metric has been previously shown to be a predictor of performance (Colmar et al., 2019; Cribbs et al., 2021; Rueda-Gómez, et al., 2023). To do so, we asked the students their level of agreement with different statements. We found that 61.1% of the students agreed with the statement “I am capable and skillful at Mathematics” (Fig. 4*A*), while 44.4% agreed with the statement “Being a good mathematics student makes me feel that my classmates and teachers think more of me” (Fig. 4*B*). These results are similar to previous reports of mathematics self-concept in university students (Rueda-Gómez, et al., 2023). However, unlike previous reports, we find that half of the students (50%) agree with the statement “In the mathematics examinations I feel unsure, desperate, and nervous” (Fig. 4*C*), while the minority of the students (44.4%) agree with the statement “My performance in mathematics largely depends on the methodology and empathy with teachers” (Fig. 4*D*). This self-concept profile is consistent with university students who are high achievers in online mathematics training (Rueda-Gómez, et al., 2023), suggesting that our students were likely to succeed in a remote PBL experiment. Finally, when asked whether they agreed with the statement “Mathematics is useful and necessary in all areas of life,” we found that 83.3% of the students agreed (Fig. 4*E*), while 88.8% of the students agreed with the statement “Mathematics is useful and necessary for a career in Biology” (Fig. 4*F*). The combination of the students’ quantitative skills and interests, as well as their self-concept consistent with high achievers in mathematics, allowed us to conclude that this cohort of students is ideal for implementing remote PBL experiments using high-density MEAs.

**Figure 5.**
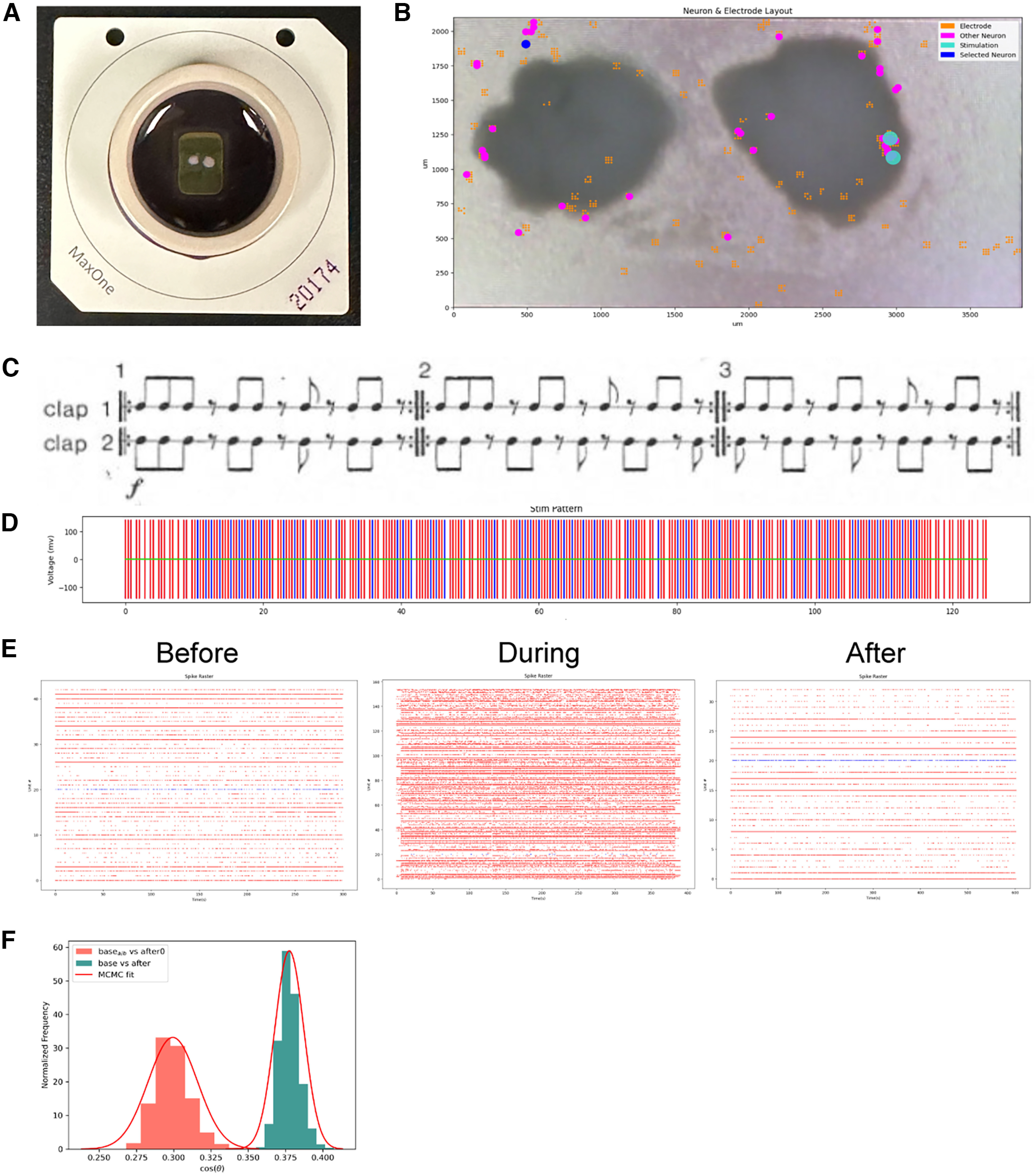
Student experiments explore programming neuronal circuits. ***A***, Students were assigned two cerebral organoids (a test and control) to use in an experiment they designed. The organoids were placed on an HD MEA, which was used to send their electrophysiology stimulus pattern. ***B***, The students used online software to explore the data. The software shows the location of neurons across the two organoids it is interacting with, as well as stimulation sites. Students can select a neuron to view specific information from it, such as the spike raster. ***C***, The experiment shown involved a team of students who decided to code the rhythm of Steve Reich’s duet, Clapping Music. Each performers’ pattern was sent to different neurons. Image is from Reich’s music score. ***D***, The students use the online software to verify that the stimulation pattern sent to the neurons follows the rhythm of the music. Red and blue represent the stimulation patterns sent to two different neuronal sites. ***E***, The students were given the neuronal spike raster data from before, during and after the experiment. These graphs display the time points at which different neurons fire action potentials. Students can determine whether the stimulus pattern they created evoked the predicted neuronal response by comparing the firing patterns between neurons. ***F***, Example of the types of computational analysis students performed. To see whether the stimulation had an effect on the neurons, this team performed a statistical test on the cosine similarity score, a metric of correlation. The test compared spontaneous activity directly after stimulation to that of 10 min after. They noticed that there was a statistically significant difference in the distribution of the cosine similarities between the two timepoints. MCMC fit = Markov-Chain Monte Carlo fit.

As part of the class, we asked students to work in groups to design and program stimulation patterns to be given to cortical organoids (Ronchi et al., 2019). They were asked to propose a hypothesis on what changes they expected to see in network behavior following stimulation. The students then analyzed the results of their stimulation patterns and presented their findings to the class. Each group was assigned two organoids: one to be used as an experimental organoid, and one control (Fig. 5*A*,*B*). To help the students conduct their electrophysiology experiment, we designed a simple application programming interface (API). Each team of students had the opportunity to watch their stimulation experiment happen on the organoid live, in real time. The experiments were hosted via Zoom, with the class TA presenting the changes in neuronal activity that occurred while the organoids were being stimulated.

The API mentioned above provided students with considerable freedom in the types of experiment they could construct. One notable group, for example, translated the rhythm of a minimalist song: Steve Reich’s Clapping Music (Fig. 5*C*,*D*). In this song, two artists clap at rhythms that synchronize and desynchronize throughout the song (Haack, 1991, 1998; Fig. 5*C*,*D*). The students wanted to see whether this syncing of stimulation patterns might yield a Hebbian-like learning outcome. This rhythm has been previously used in mathematics problems in combinatorics and group theory (Haack, 1991, 1998). The students translated each of the clapping sequences to an electrode stimulation pattern using the API (Fig. 5*D*). These patterns were then applied to the experimental organoid (Fig. 5*E*) and the students found that the stimulation pattern had a measurable effect on neuronal behavior (Fig. 5*F*).

**Figure 6.**
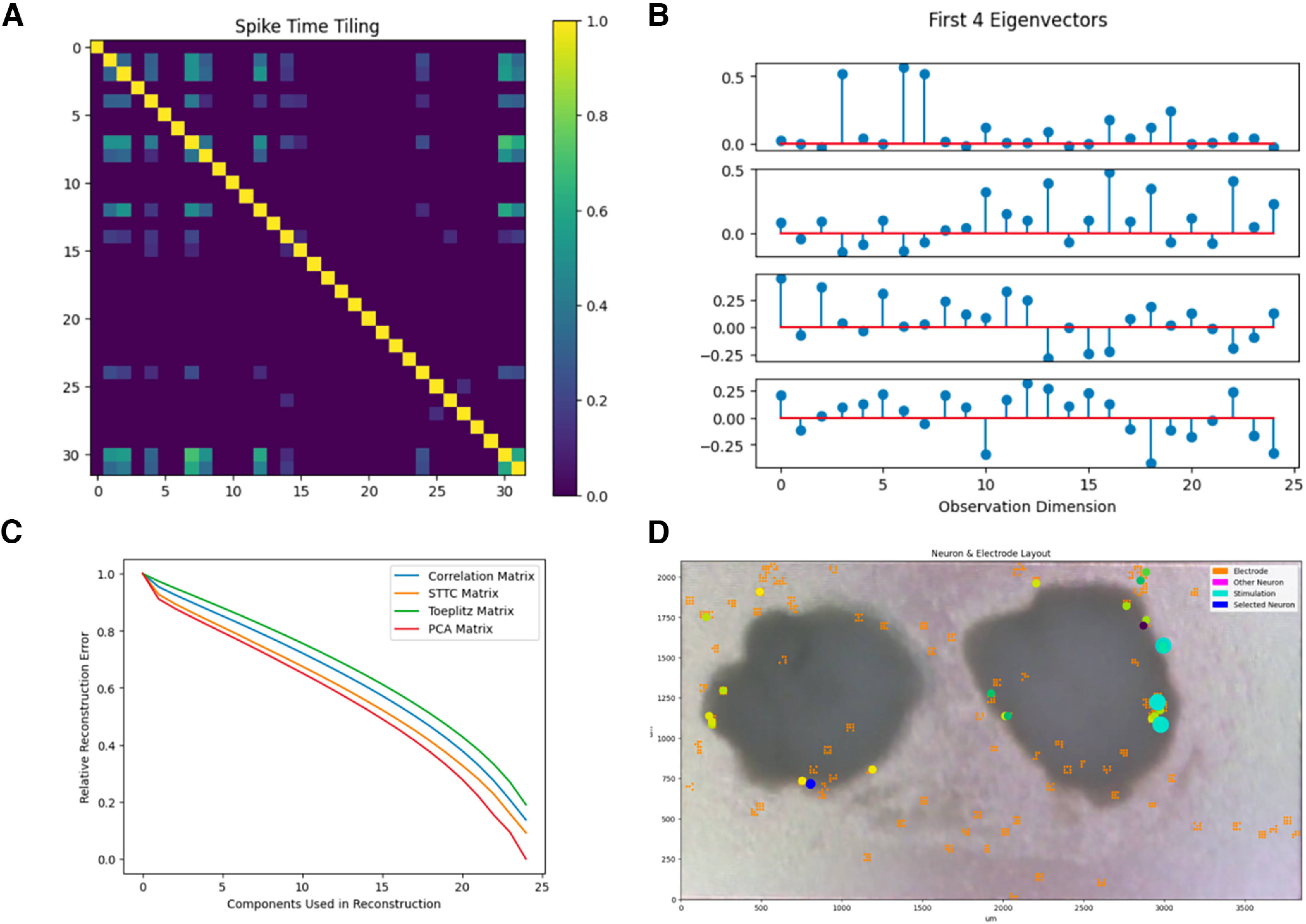
Example of Mathematics of the Mind student-project applying tetanic pattern to cortical organoids. ***A***, Spike time tilling matrix for the electrophysiology data recorded from cortical organoids. ***B***, Eigendecomposition on the spike time tilling matrix to extract its eigenvalues and eigenvectors. For illustration, the first four eigenvectors are plotted. ***C***, Comparison of eigendecomposition on the spike time tiling matrix to other popular correlation techniques. Specifically, they considered how the method’s reconstruction error compared with that of performing principal components analysis on the correlation matrix. ***D***, Superimposition of the first eigenvector’s values (represented as small circles in the blue-yellow range) on top of their corresponding neural unit allowed the students to differentiate which neurons came from which organoid.

The other groups created experiments of similar rigor and creativity (Fig. 6). Three of the five groups stimulated organoids with a high frequency tetanic pattern (Tamura et al., 2020). This experiment was popular because it was explained to have a high chance of prominent changes. Each group approached the problem using a unique protocol they designed. One group used the freedom that the stimulation API provided to design an experiment which generated a random walk over stimulation amplitudes, producing a unique sequence during each generation. This group was interested in whether these stimulation sequences produced unique patterns between neurons connected in a circuit.

The students were told to rigorously analyze the resulting data from their experiment to determine whether the experiment’s hypothesis was correct through techniques they were taught throughout the class: correlation matrix, spike time tiling coefficient, interspike intervals, and interneuronal spike latencies. Most importantly, the students had to implement some form of original analysis not taught during the class. This could either be a method learned from reading literature, or a novel technique they design themselves. Most groups implemented either a basic statistical test to verify their hypothesis, or an unsupervised machine learning technique to help interpret results. However, some groups developed novel analysis methods. Of note, one group implemented the underlying algorithm used in principal components analysis (PCA) but using the spike time tiling matrix as the input into the algorithm (Fig. 6*A–D*). The group showed that this new technique was able to discern the test from control organoid based on neural signals (Fig. 6*D*).

### Organoid experiments lead to higher interest in stem cell and neuroscience research in mathematics students

**Figure 7.**
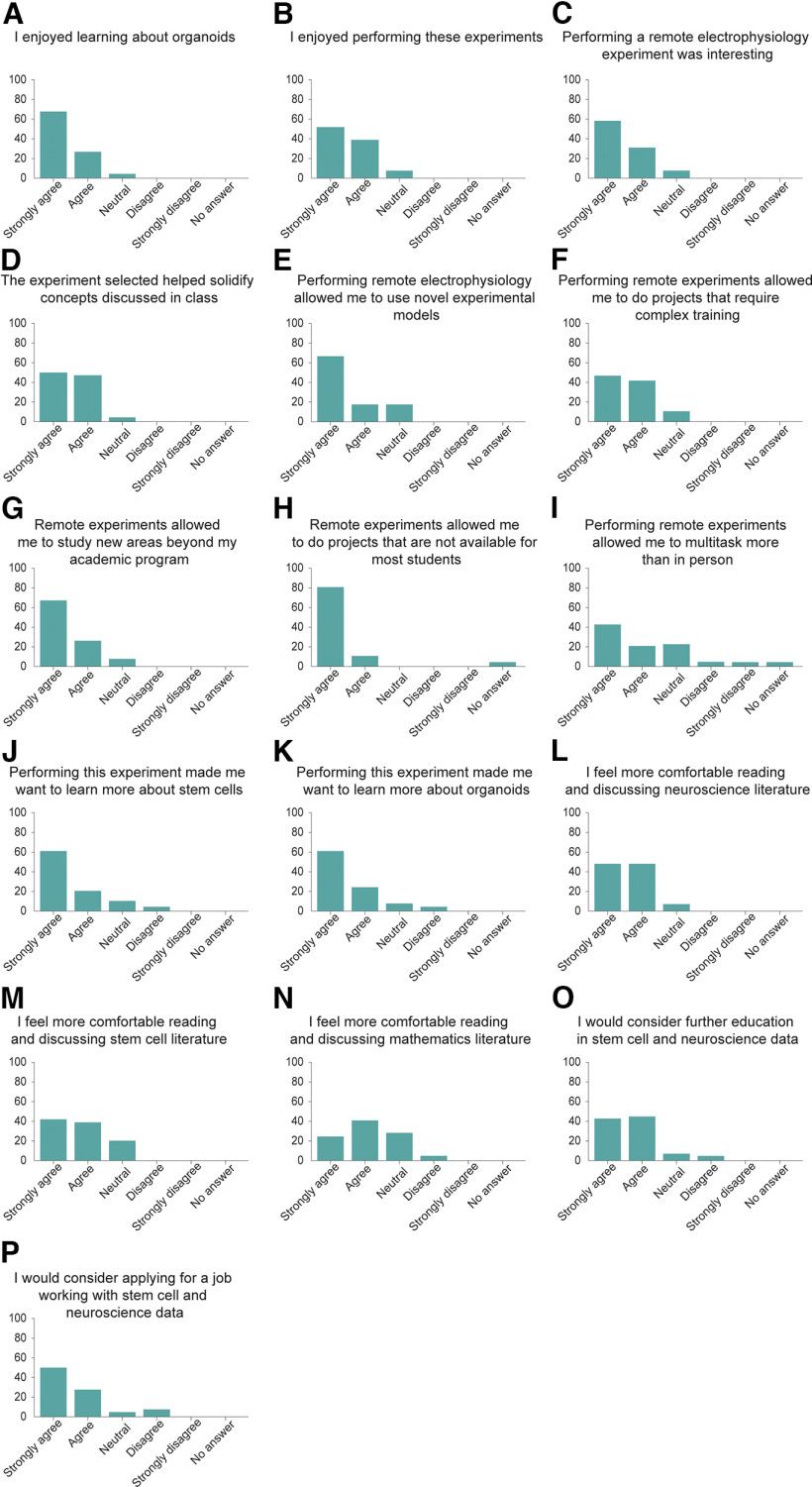
Mathematics students report positive attitudes toward stem cells and neuroscience topics after performing experiments with cortical organoids. ***A–P***, Postexperiment survey results for the Mathematics of the Mind course. *n* = 24 students. All of the 11 survey questions were statistically significant, using a one-sample two-tailed *t* test (*p*-value less or equal to 0.05). *y*-axis represents percent of students.

To understand how the integration of HD MEA recording and stimulation of neuronal cell cultures in the classroom affects the students’ interest in neuroscience and stem cell research, we surveyed the Mathematics of the Mind students immediately after their presentations. All 24 students enrolled in the class responded to the survey. We found that 95.8% of the students said that they enjoyed learning about organoids (Fig. 7*A*), and 91.6% enjoyed performing these experiments (Fig. 7*B*). In addition, we found that 91.6% of the students thought that performing remote electrophysiology experiments was interesting (Fig. 7*C*), while 95.8% of the students thought that the experiment selected helped solidify the concepts discussed in the class (Fig. 7*D*).

We then asked the students for their perceptions after performing remote electrophysiology experiments. We found that 83.3% of the students thought that performing these experiments allowed them to perform novel and complex experiments (Fig. 7*E*), while 87.5% of the students thought that this approach allowed them to do experiments that required complex training (Fig. 7*F*). 91.6% of the students thought that these experiments allowed them to study new areas beyond their academic program (Fig. 7*G*). A total of 95.8% of the students considered that performing remote experiments allowed them to do projects that are not available to most students around the world (Fig. 7*H*), while 62.5% of the students thought that performing remote experiments allowed them to multitask more than in person labs (Fig. 7*I*).

Then, we focused on understanding how performing these experiments influenced the students’ interest in neuroscience and stem cell research. We found that 83.3% of the students thought that performing these experiments increased their interest in stem cells (Fig. 7*J*), while 87.5% reported an increased interest in organoids (Fig. 7*K*). Remarkably, we also find that after this course most of the students report a higher comfort in reading and discussing scientific literature in neuroscience (91.6%; Fig. 7*L*), stem cells (79.1%; Fig. 7*M*), and mathematics (66.6%; Fig. 7*N*).

Finally, we asked the students how this work affected their career prospects. We found that 87.5% of the students would consider continuing further education in stem cells and neuroscience (Fig. 7*O*) and 79.1% would consider applying to jobs analyzing stem cell and neuroscience data (Fig. 7*P*). Altogether we conclude that our approach positively affected the students’ interest in stem cell and neuroscience.

### Internet-connected organoids lead to equivalent outcomes in multiple courses

Given that the students in both the Techniques in Tissue Culture and the Mathematics of the Mind courses reported positive feelings toward topics in stem cells and neuroscience after the completion of the activities, we compared the postcourse survey answers of both courses. In total, 8 questions were shared between both postcourse surveys. These included questions that target the students’ experience, their interest in the topic and their desire to gain further knowledge in stem cells and neuroscience topics (Table 1). We find no statistical difference between any of the answers from students in either course, suggesting that approaches that Internet-connected organoids are effective tools for both courses.

**Table 1.** Comparison of postcourse surveys between Techniques in Tissue Culture and Mathematics of the Mind courses

Question	*p*-value	Significance
I enjoyed learning about organoids	0.56	No
I enjoyed performing these experiments	0.36	No
Performing a remote [microscopy/electrophysiology] experiment was interesting	0.53	No
Performing remote experiments allowed me to multitask more than in person labs	0.90	No
The experiment selected helped solidify concepts discussed in class	0.68	No
Performing remote [microscopy/electrophysiology] allowed me to use novel experimental models	0.85	No
Performing this experiment made me want to learn more about stem cells	0.96	No
Performing this experiment made me want to learn more about organoids	0.85	No

## Discussion

The impact of regenerative biology in the biotechnology and translational medicine sectors has grown exponentially over the past decades (Jacques and Suuronen, 2020). Therefore, there is an increased need for exposing students and young professionals to topics related to stem cells and regenerative medicine, including training in experimental design and applications of the technology (Wyles et al., 2019; Sterner et al., 2020; W Sandoval et al., 2022). Scalable approaches that can keep students motivated and engaged during their training should be prioritized to fulfill this demand (Wyles et al., 2020). The use of Internet-enabled platforms can inexpensively scale the number of targeted students and reach traditionally underserved communities (Findyartini et al., 2021; Baudin et al., 2022b). Indeed, the use of virtual programs in the topic of regenerative medicine have been explored, especially during the COVID-19 pandemic (Wyles et al., 2020). Yet, in passive online teaching approaches maintaining students’ interest is challenging (Gares et al., 2020), and as a result, virtual courses often have a high dropout rate (W. Wang et al., 2023).

PBL approaches in STEM are effective at retaining students, especially those of underrepresented backgrounds (Ferreira et al., 2019). PBL courses are particularly successful when they integrate themes that are trendy in society (Cotner and Gallup, 2011; Carosso et al., 2019). To this end, the high interest of the public and popular media in brain organoids (Presley et al., 2022) can make them ideal tools to train the next generation of students in regenerative medicine. Here, we took advantage of cortical organoids to design PBL modules and target students both in the life sciences, as well as other STEM disciplines, including mathematics, physics, engineering, and computer science. Both groups of students reported that performing the experiments in organoids helped solidify the concepts discussed in class. Furthermore, we showed that this approach increased the interest in topics related to neuroscience and stem cell research of both groups of students. In addition, nonbiology students showed a higher interest in continuing their education in these topics. Moreover, the Internet connectivity of our laboratory hardware allowed the students to perform the experiments remotely, up to 125 km from the experimental site. Additionally, this Internet connectivity enables us to scale the number of users at a marginal price (Ly et al., 2021; Baudin et al., 2022b). These two properties open the possibility of massively deploying cloud-enabled technologies for training in neuroscience and regenerative biology.

We piloted this approach by adding a cortical organoid-based remote experiment module to two courses: a cell culture techniques and a mathematics course. However, organoids can be used in a variety of other PBL courses. For example, cortical organoids of multiple species (Pollen et al., 2019; Mostajo-Radji et al., 2020) can easily be integrated in an evolutionary biology course. “Assembloids,” fused organoids of different brain regions (Paşca, 2019), can be used to train students in systems neuroscience. Similarly, connectoids (Cullen et al., 2019; Kirihara et al., 2019; Adewole et al., 2021), can be used in the context of teaching higher level interactions between brain areas. Moreover, organoids can become important tools to teach chemistry in the context of drug screens (Schuster et al., 2020; Salick et al., 2021). Bioengineering courses can benefit from organoids experiments to teach biomaterials and sacrificial networks (Grebenyuk and Ranga, 2019). Finally, deriving organoids from multiple body regions beyond the brain, could be of benefit to physiology and pharmacology courses (Clevers, 2016).

While the work presented here is the proof of principle in two undergraduate courses, it can easily scale to larger amounts of users. For example, with microscopy-based experiments, larger classes can form subgroups of students who work together, multiplex experiments through time scheduling, and poll students to vote for specific classroom-wide experiments. Importantly, because the experiments are streamed through YouTube, there is no technical limit in the number of users (Pires and Simon, 2015). Furthermore, because YouTube takes advantage of dynamic adaptive video streaming technology (Suman et al., 2022), it can deliver a similar quality of experience to students located in poor network conditions (Seufert et al., 2015). Indeed our group has previously applied these strategies in multicourse projects scaling to over 130 students and five countries simultaneously, showing that this approach can develop STEM identity and transmit complex topics in students in all the settings tested (Baudin et al., 2022b).

The scalability of electrophysiology projects would follow a different approach. While the HD MEAs used in these experiments have a high cost ($200/chip) and require expensive capital equipment, there are ∼26,000 electrodes in the HD MEAs, which can be distributed to lower the per user costs. Specifically, users can read from 1024 electrodes simultaneously and of those they can stimulate from (Ronchi et al., 2019). We can switch to a new set of 1024 in seconds. This allows us to multiplex the system in various ways: by assigning different sets of electrodes for recording and stimulation to different subgroups within the class or individually to each student, depending on class size. For extended experiments, each student or group can design a stimulation pattern and our interface can apply these extended patterns to the same or different chips sequentially. In addition to the multiplexing of experiments, we have developed a strategy to scale the use of the software and lecture materials to reach more students. The coding lectures and homework were aggregated into a single public repository for future education (https://github.com/Braingeneers-Education). We integrated a newly developed cloud environment, Github Codespaces, to allow anyone to access course tutorials. The software is web based, requiring no downloads, and can be run from any computer or tablet.

We showed that the integration of cortical organoids to the classroom develops interest in stem cell and neuroscience topics. However, this study has some limitations. First, the work was performed in a small number of students, and larger studies including multiple classrooms from around the world are necessary to understand how the local context affects students’ experience. Second, while the focus of this study was on measuring the user experience of students after the introduction of remote cortical organoids experiments, future research is needed to understand the potential of organoids as pedagogical tools to transmit complex neuroscience and stem cell concepts. In the past, we used remote tissue culture as a viable alternative to in person training in underserved locations and have shown that this approach is an effective pedagogical tool (Baudin et al., 2022b). However, controlled experiments comparing multiple courses throughout years are still needed to understand the true potential of organoids in the classroom. Third, our interventions were short and students were surveyed soon after the experiments were concluded. Many of the students graduated after the course. Therefore, it was difficult to track the students long-term. We are currently working on courses that would introduce students to cortical organoids early in their undergraduate degrees, which will enable us to measure the long-term effects of these interventions. This is particularly relevant to remove the “novelty effect” from analysis. Indeed, other studies using computer-based technology, such as virtual reality platforms, in PBL have shown the continued student engagement long after the introduction of these technologies (Tsay et al., 2020). This is likely because of the “familiarization effect” as students become more accustomed to the technologies (Rodrigues et al., 2022). Finally, while the experiments can be performed in poor network conditions, they still require Internet connectivity, which can limit the accessibility of students in remote locations. New partnerships that leverage engineering innovations, such as tethered balloons or drones, to deliver Internet (Alsamhi et al., 2016; Belmekki and Alouini, 2022) can become crucial to expand the reach and impact of this work.

In conclusion, we provided a proof of principle study using organoids as novel pedagogical tools for undergraduate education. We developed PBL-based curricula using imaging and electrophysiological tools. By connecting these technologies to the cloud, we were able to teach the courses remotely, while at the same time scaling to allow multiple students to interact with the same experimental platform. This approach led to a higher interest of the students toward stem cell and neuroscience paths. Altogether, this approach has the potential to greatly expand training in regenerative medicine and neuroscience and reach students currently underserved in their communities.

## Synthesis

Reviewing Editor: Mark Laubach, American University Decisions are customarily a result of the Reviewing Editor and the peer reviewers coming together and discussing their recommendations until a consensus is reached. When revisions are invited, a fact-based synthesis statement explaining their decision and outlining what is needed to prepare a revision will be listed below. The following reviewer(s) agreed to reveal their identity: Zoltan Simandi.

The reviews of your manuscript are below. Please revise to address all points that were raised in your resubmission. Thank you for submitting your work to eNeuro!

Reviewer 1

I found this manuscript to be quite fascinating and important as it delves into the ways in which cloud-based technologies can facilitate STEM resource sharing, and how this can benefit underserved communities through knowledge sharing and outreach initiatives involving pluripotent stem cell-derived organoid advancements. While I am excited about seeing this manuscript published as soon as possible, I have two minor concerns that I would like to highlight:

1) I believe it would be worth considering the integration of statistical analysis when discussing the data on the students’ answers. However, I understand that this might be beyond the scope of this work.

2) It would be great to see more details included in the discussion about the generalizability of the findings to other educational settings, as well as a more nuanced exploration of future directions for implementing this approach on a wider scale in research and education in this area.

Reviewer 2

This pilot program underscores the potential of internet-enabled technologies to democratize access to STEM education, particularly in the field of stem cell research.

While the approach described in the text appears promising in its intent to foster diversity and inclusion in STEM education, there are several weaknesses that warrant consideration and more discussion:

The text mentions the utilization of internet-enabled technologies as a means to bridge the educational gap. However, it does not address the potential limitations in access to these technologies. Underserved students might not have access to the required computers, or other devices, which could exclude them from participating in these remote experiments.

There is a lack of detail regarding the practicality and cost-effectiveness of implementing such equipment in educational settings. Scaling such a program to accommodate a more significant number of students and institutions is a critical consideration.

Without a comprehensive assessment, it is challenging to determine the long-term impact and effectiveness of this approach.

It’s crucial to examine whether remote experiments, while innovative, adequately fulfill the learning objectives and pedagogical goals of STEM education. Are students gaining a deep understanding of the subject matter, or are they merely participating in remote experiments for the sake of novelty? The text does not provide insights into the pedagogical effectiveness of this approach.
